# Modulation of microglial phagocytosis via the GAS6-MERTK pathway regulates pathological angiogenesis in the mouse oxygen-induced retinopathy model

**DOI:** 10.1038/s41419-025-07744-4

**Published:** 2025-06-02

**Authors:** Canelif Yilmaz, Irina Korovina, Anke Witt, Farid Abdallah, Bianca Müller, Carmen Hentsche, Anika Fleischhauer, Stephan Speier, Andreas Deussen, Anne Klotzsche - von Ameln

**Affiliations:** 1 Institute of Physiology, Faculty of Medicine, Technische Universität Dresden, Dresden, Germany; 2 Institute for Clinical Chemistry and Laboratory Medicine, Faculty of Medicine, Technische Universität Dresden, Dresden, Germany; 3 OncoRay, National Center for Radiation Research in Oncology, Faculty of Medicine, Technische Universität Dresden, Dresden, Germany

**Keywords:** Cell death and immune response, Retina

## Abstract

Ischemic retinopathies (IR) are major causes of blindness worldwide. They are characterized by an exuberant hypoxia-driven pathological neovascularization (NV). While it is well accepted that immune cells contribute to both physiological and pathological retinal angiogenesis, our knowledge of various processes and underlying mechanisms, especially in the direct interaction with endothelial cells (EC), is still very limited. Here, we addressed the role of microglial phagocytosis of apoptotic EC in the context of pathological hypoxia-related NV in the mouse oxygen-induced retinopathy model (OIR). We utilized endothelium-specific fluorescent reporter mice to study the kinetics of EC phagocytosis by leukocytes in OIR. Indeed, we observed phagocytic microglia in close proximity to the pathological vessels and an altered phagocytosis rate by flow cytometry compared to controls. We observed a decrease in the phagocytic rate in early hypoxia-driven stages of OIR, whereas in later stages where pathological vessels appear, the phagocytosis rate was increased. Myeloid-specific deletion of the suppressor of cytokine signaling protein 3 (SOCS3) was previously shown to induce increased phagocytic activity due to overexpression of the opsonin molecule growth arrest-specific 6 (GAS6). In myeloid SOCS3-deficient mice, we observed a reduction of pathological NV in OIR. This reduction could be reversed by neutralizing GAS6 via administration of recombinant MERTK protein, the receptor for GAS6 expressed on myeloid cells. Furthermore, exogenous GAS6 supplementation increased microglial phagocytosis in vitro and limited pathological NV in OIR. Our data suggest that the promotion of immune cell phagocytosis by the modulation of the GAS6-MERTK axis might represent a potential target for the treatment of pathological NV in IR.

## Introduction

The most important forms of IR are retinopathy of prematurity (ROP) and diabetic retinopathy (DR) [1–3]. Their common feature in the end stage of the disease, called proliferative retinopathy, is hypoxia-triggered pathological NV. This over-compensatory, dysregulated NV leads to the development of malformed, fragile, and leaky blood vessels (= tufts), which are ectopic and can invade the vitreous cavity, which can further aggravate the disease, resulting in vision impairment and blindness [1]. In recent years, immune cells, especially myeloid cells, that include retinal microglia and macrophages, have emerged as crucial players in maintaining retinal homeostasis and exert diverse angiogenic functions [1, 4–8]. In IR, the delicate retinal homeostasis is disturbed, which is sensed by myeloid cells, especially microglia, resulting in their activation. This involves changes in their proliferation, migration, morphology, cytokine and growth factor secretion, as well as alterations of their phagocytic activity [1, 4, 9]. Phagocytosis, the elimination of dying/ dead cells and cellular debris, is an essential process to maintain and restore tissue integrity, which supports tissue homeostasis [10, 11]. This is especially important, as multiple pathological situations, including DR and ROP, are associated with the rapid accumulation of cellular detritus and cell death in ECs [12, 13]. In line in the mouse OIR model, particularly in the pathological vessels, many apoptotic ECs were found [14, 15]. Interestingly, in ROP patients, pathological tufts can be spontaneously cleared, resulting in normalization of the retinal vessels and subsequent healing [16–18]. Based on this state of knowledge, we addressed the question, whether immune cell phagocytosis is implicated in the removal of altered/ dying ECs in proliferative retinopathies and whether this mechanism is part of the pathophysiology of IRs.

Previously, we have shown that SOCS3-deficient mononuclear phagocytes had an increased expression of *Gas6*, an opsonin molecule linking the phosphatidylserine (PS) found on the surface of apoptotic cells to the phagocytic receptor MERTK, functioning as an “eat me” signal. Elevated expression of *Gas6* resulted in enhanced phagocytosis of apoptotic retinal ECs, and furthermore limited the angiogenic sprouting of aortic rings [19]. In addition, blocking of GAS6 with a MERTK-Fc chimera protein not only diminished phagocytosis of apoptotic ECs but also abrogated the reduced angiogenic sprouting in SOCS3-deficient aortic rings. To test the relevance of immune cell phagocytosis on pathological angiogenesis in vivo, we first subjected a Flk1:Cre/tdTom^fl/fl^ transgenic reporter mouse line to the OIR model to prove whether apoptotic/ dying ECs in the retina are phagocytized and subsequently eliminated by myeloid cells. With flow cytometry experiments, we found indeed an uptake of red-fluorescent EC particles in retinal myeloid cells in the OIR model, as well as in mice kept in room air. Surprisingly, at early stages before the onset of pathological NV (P12 and P15) the phagocytosis rate of ECs was decreased in the OIR model compared to controls, while at later stages (P17 and P21), the phagocytosis rate was elevated. Thus, we hypothesized that compensating for the observed drop of immune cell phagocytosis at early stages in OIR, e.g., by the induction of the GAS6-MERTK axis, might attenuate the angiogenic response and thus limit pathological NV. To this end, we engaged mice with myeloid SOCS3-deficiency, with an increased phagocytosis rate [19, 20], in the OIR model [1, 18, 21]. We found that myeloid SOCS3-deficiency remarkably decreased pathological NV without effects on physiological retinal vessel formation. Neutralization of GAS6 with a recombinant GAS6 receptor MERTK reversed the inhibition of NV in SOCS3-deficient mice. For an additional “proof of principle” wild type (WT) mice in the OIR model received an intravitreal injection of recombinant GAS6, resulting likewise in decreased pathological NV. These findings not only demonstrate that a drop/ alteration in the phagocytosis rate of myeloid cells might contribute to an elevation in pathological NV, but also indicate that the modulation of immune cell phagocytosis, e.g. by the modulation of the GAS6-MERTK axis, might represent a new approach for the therapy of IR.

## Results

### Microglial activation and phagocytosis of ECs is observed in the OIR model

**Fig. 1:**
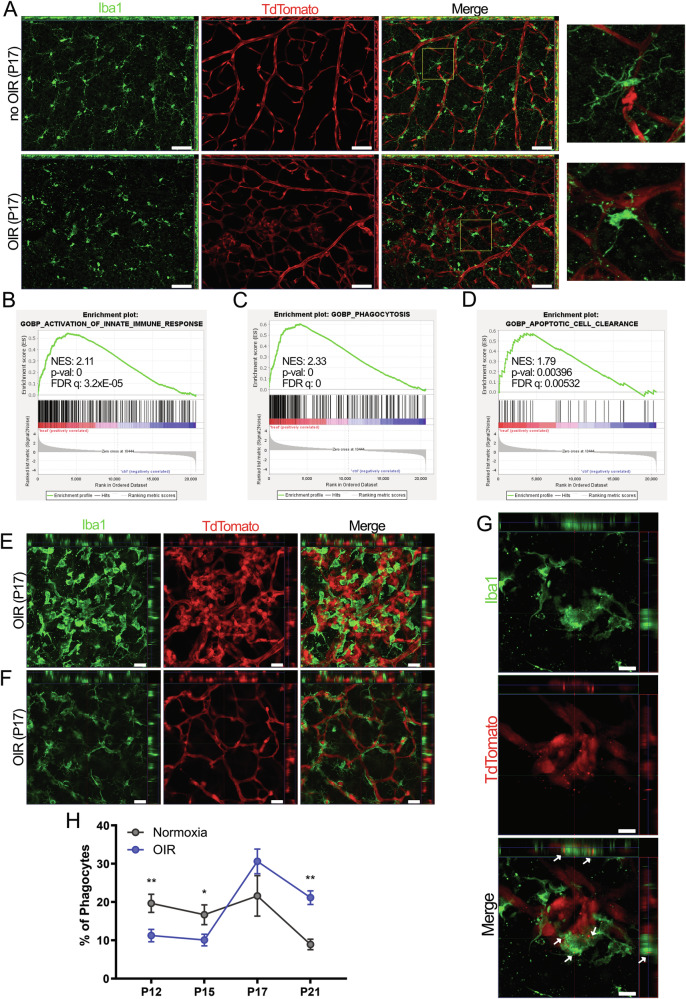
Microglial activation and phagocytosis in the mouse OIR model. **A**, **E–G** Flk1:Cre/tdTom^fl/fl^ mice were subjected to the OIR model, or kept in room air. At P17, pups were sacrificed, and retinal whole mounts were immunostained for IBA1. **A** Wide field images of retinal whole mounts from OIR and non-OIR mice. Maximum intensity projection of 10 z-stacks from the SVP layer were created. Images show differences in microglial morphology. Scale bar: 50 µm. **B–D** Bulk RNA sequencing was performed on microglial cells (CD11b^+^) MACS sorted from C57BL/6 J mice subjected to the OIR protocol, or control littermates kept in room air, and sacrificed at P17. (*n* = 3 mice per group). GSEA results are shown for “Activation of Innate Immune Response” (**B**), “Phagocytosis” (**C**), and “Apoptotic Cell Clearance” (**D**) gene sets. Confocal image of a retinal whole mount from mouse with OIR, demonstrating differences in vascular and microglial morphology in SVP (**E**) and IVP (**F**) layers. Scale bar: 20 µm. **G** High magnification confocal image of a retinal whole mount from mouse with OIR. Arrows showing red endothelial particles engulfed by an IBA1^+^ cell. Scale bar: 10 µm. **H** Flk1:Cre/tdTom^fl/fl^ mice subjected to OIR, or kept in room air, were sacrificed at P12, P15, P17, and P21, and flow cytometry was performed on retinal cell suspensions to assess the percentage of phagocytic microglial cells (CD45^+^tdTom^+^). The frequency of phagocytic microglia is shown as a % of CD45^+^ microglial cells. (*n* = 6–11 mice at P12, *n* = 10–11 mice at P15, *n* = 6–11 mice at P17 and *n* = 3–5 mice at P21). Data are presented as mean ± SEM, *p < 0.05, **p < 0.01.

Initially, we wanted to address the role of microglia during the progression of OIR. For this purpose, we have utilized Flk1:Cre/tdTom^fl/fl^ mice, which express the red fluorescent tdTomato protein in ECs, subjected them to the OIR protocol, and sacrificed at P17, a point associated with the peak of pathological angiogenesis. Immunofluorescence staining against IBA1 on retinal whole mounts revealed that microglia, in retinas isolated from mice maintained in room air had small cell bodies, with elongated dendritic processes, typical of resting state microglia [22]. Conversely, microglia from mice subjected to the OIR protocol displayed an ameboid morphology, with larger cell bodies and fewer dendritic extensions, indicating microglial activation [1, 23] (Fig. 1A). In order to confirm microglial activation, and to identify which cellular processes are enhanced in OIR microglia, we performed bulk RNA sequencing on MACS sorted microglia from WT mice subjected to the OIR protocol, and control littermates kept in room air at P17. A total of 1223 differentially expressed genes (DEG’s) were identified at FDR 1, of which 1048 were upregulated, while 175 were downregulated (Fig. S1A). Gene set enrichment analysis (GSEA) performed on the ranked list of DEG’s using the MSigDB GO:BP collection revealed that “Activation of innate immune response” gene set (Fig. 1B), as well as “Leukocyte proliferation” and “Leukocyte migration” gene sets (Fig. S1B, C) were significantly enriched in microglia sorted from OIR mice, confirming microglial activation. Furthermore, “Phagocytosis” and “Apoptotic Cell Clearance” gene sets were also significantly enriched in microglia sorted from OIR mice, suggesting that microglia can perform phagocytosis of cells in the OIR model (Fig. 1C, D). Additionally, gene sets “Positive regulation of EC proliferation,” “Regulation of blood vessel EC migration” and “Regulation of vasculature development” were positively enriched in OIR microglia (Fig. S1 D–F). We also found increased expression of secreted factors such as *Vegfa*, *Igf1*, *Fgf2*, and Angiopoietin-2, (Fig. S1 G), which indicates that microglia are capable of performing various functions in the regulation of angiogenesis [1, 8, 9].

We next assessed spatial localization of microglia in the retinas by utilizing Flk1:Cre/tdTom^fl/fl^ mice subjected to the OIR protocol, sacrificed at P17. In line with previous publications [24, 25], we observed that microglia in the OIR retinas were localized in close proximity to the vasculature, especially in the pathological tufts. Furthermore, microglia in the superficial vascular plexus (SVP) layer, where abnormal vasculature and epiretinal tufts are found, had an ameboid morphology indicating microglial activation (Fig. 1E), whereas microglia in the intermediate vascular plexus (IVP) layer showed ramified morphology, maintaining their resting state (Fig. 1F). Further analysis by confocal imaging revealed red fluorescent particles engulfed by IBA1+ microglial cells within the epiretinal tufts (Fig. 1G), supporting the idea that microglia perform phagocytosis of ECs in IR.

Subsequently, we attempted to evaluate the phagocytic activity of retinal microglia throughout the course of OIR. To this end, we have performed flow cytometry analysis of retinas isolated from Flk1:Cre/tdTom^fl/fl^ mice subjected to the OIR protocol or maintained at room air, at P12, P15, P17, and P21 (Fig. 1H). Our results showed that the percentage of phagocytic microglia (tdTom^+^ CD45^+^ cells) in mice kept in room air was relatively stable throughout physiological retinal development between P12-P17, and declined at P21. Interestingly, compared to corresponding controls, OIR mice had fewer phagocytic microglia at P12 and P15 and, in contrast, phagocytosis was enhanced 3-fold at later stages of OIR progression at P17 and P21. In addition, we observed a lower amount of EC in OIR compared to no OIR at P12, a similar amount of ECs at both conditions at P15 and P17, and a higher number of ECs in OIR at P21 compared to no OIR (Fig. S1H). Together, we demonstrated that microglial activation is observed in the OIR model, and microglia perform phagocytosis throughout both healthy and pathological vascular development. Thus, we hypothesized that the reduction of phagocytosis observed in the early stages (P12 and 15) of OIR, in cooperation with the increased expression of proangiogenic factors, may lead to the excessive vascular growth, and formation of pathological tufts, hence establishing the therapeutic potential of targeting phagocytosis in IR.

### *Gas6* expression is regulated through the course of OIR

**Fig. 2:**
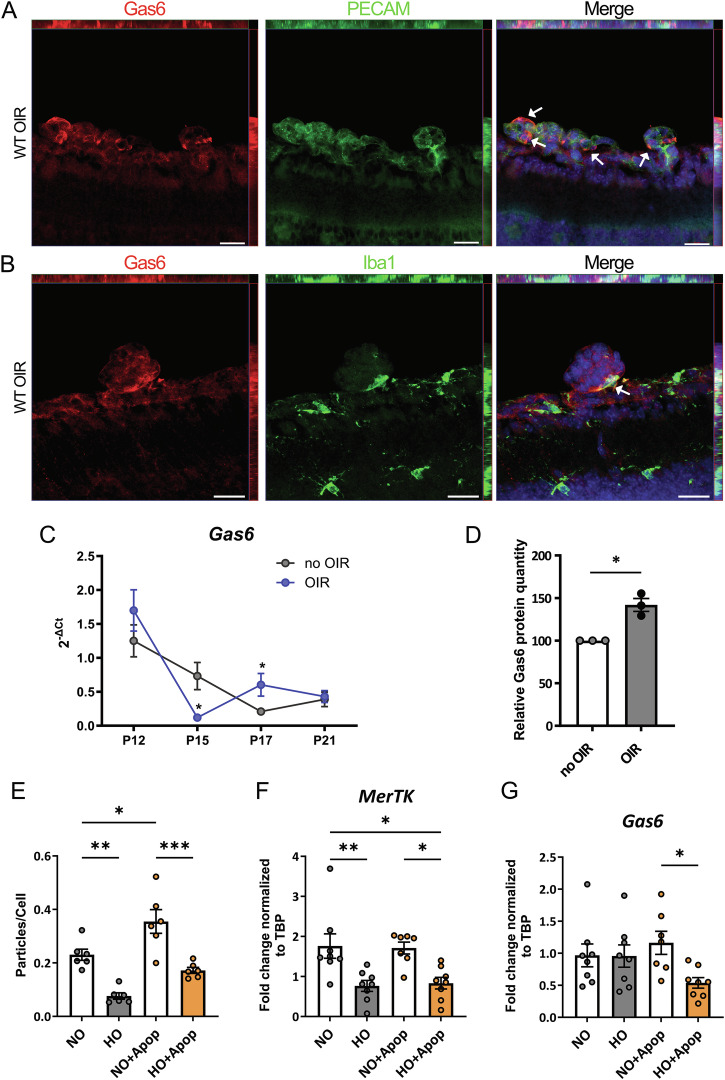
GAS6 expression in the OIR model. **A**, **B** C57BL/6J mice were subjected to the OIR model, or kept in room air. At P17, pups were sacrificed, and retinal cross sections were immunostained for GAS6 combined with PECAM-1 or IBA1, and the nuclei were counterstained with DAPI. Wide field images of co-localization of GAS6 (red) with ECs (PECAM-1, green) (**A**), or with microglia cells (IBA1, green) (**B**) in the pathological tufts (white arrows). Scale bar: 20 µm. (**C**) Analysis of *Gas6* mRNA expression in retinas of C57BL/6J mice subjected to the OIR protocol at indicated time points (mice maintained in room air were used as control). Relative expression to *Tbp* without normalization is shown. (*n* = 4–5 mice). **D** Graph illustrating GAS6 protein expression in retina lysates of C57BL/6J mice subjected to the OIR model or controls kept in room air at P17. Intensity of GAS6 signal in non-OIR group was set as 100 for each experiment. *n* = 3, retinas from 3–4 mice of one breeding were pooled for one sample. **E** Primary microglia were cultured with or without apoptotic HUVECs under normoxia or hypoxia for 24 h. Phagocytosis rate was quantified 90 minutes after addition of pHrodo labeled apoptotic HUVECs. (*n* = 6 microglia cell isolations) (**F**, **G**) *Mertk* and *Gas6* mRNA expressions in primary microglia cultured with or without apoptotic HUVECs under normoxia or hypoxia (1% O_2_), for 24 h. NO normoxia, HO hypoxia, Apop:apoptotic HUVECs. *n* = 7–8 microglial cell isolations. Data are presented as mean ± SEM, *p < 0.05, **p < 0.01, ****p < 0.0001.

As our previous research has revealed that increased GAS6 production by microglia induced phagocytosis [26], we selected GAS6 as a target for further investigations. Initially, we characterized the expression and tissue distribution of GAS6 in the OIR model. We performed immunofluorescent staining for GAS6 together with PECAM-1 (Fig. 2A) or IBA1 (Fig. 2B) on retinal tissue sections from P17 WT mice subjected to OIR protocol. Our data revealed that GAS6 is found on epiretinal neovascular tufts, most abundantly on EC membranes, and at EC-microglia interfaces. This suggested that GAS6 performs its function as an opsonin by labeling pathological ECs for phagocytic recognition by microglia. Following, we analyzed *Gas6* expression in retinas isolated from WT mice subjected to OIR protocol, or kept in room air, at P12, P15, P17, and P21. Interestingly, we revealed that retinal *Gas6* expression was significantly lower at P15 in OIR retinas compared to healthy controls, and this difference was reversed at P17. Notably, at P12 and P21 both the OIR mice and room air mice had similar levels of *Gas6* expression in the retina (Fig. 2C). A cytokine array performed with whole retina lysates confirmed that at P17, GAS6 protein was produced significantly more in retinas of OIR mice compared to room air mice (Fig. 2D). In order to identify whether the altered *Gas6* expression in the retina originates from microglial *Gas6* expression, we MACS sorted retinal microglia at different time points in OIR and control mice. However, *Gas6* expression was not altered between OIR and control mice at the different time points (Fig. S2A), indicating that changes in microglial *Gas6* expression might not be the main source of the altered *Gas6* levels in the OIR. Interestingly, we observed a clear induction of *Vegfa* expression in retinal microglia sorted from OIR mice compared to healthy controls, indicating that these cells contribute to the hypoxic induction of *Vegfa* in the course of OIR (Fig. S2B). Next, we tried to identify which physiological or pathological stimuli may contribute to the different phagocytosis rates observed in OIR mice compared to controls. Our in vitro phagocytosis assay revealed that microglia cultured under hypoxic conditions showed impaired phagocytic activity in comparison to microglia cultured under normoxia, both when preincubated with apoptotic HUVECs or not (Fig. 2E). Interestingly, preincubation with apoptotic HUVECs alone was able to increase phagocytic activity of microglia, suggesting that the factors produced by apoptotic HUVECs, or the uptake of apoptotic HUVECs enhances the phagocytic activity of microglia, while hypoxia has an inhibitory effect. Following, we questioned whether the functional changes in microglial phagocytosis might be explained by changes in expression of important phagocytosis molecules. To this end, we utilized primary mouse microglia cultured under hypoxia (1% O2) or normoxia (room air), or in contact with apoptotic HUVECs, or both. Gene expression analysis performed on microglia subjected to aforementioned pathological stimuli showed that hypoxia alone was sufficient to decrease the expression of the GAS6 receptor *MerTK* in primary microglia (Fig. 2F). In contrast, *Gas6* expression was not altered under hypoxic conditions, only together with the presence of apoptotic ECs *Gas6* expression was reduced (Fig. 2G). *Vegfa* and *Glut1*, well-described hypoxia-regulated genes, confirmed sufficient hypoxia treatment in the experiment (Fig. S2C, D).

Together, our results suggest that GAS6 plays a role during OIR progression, presumably by opsonizing apoptotic ECs for recognition and elimination by phagocytes. Furthermore, we showed that gene expression levels of *Gas6* change through the course of the OIR. Additionally, we have determined hypoxia to be an effective stimulus, which lead to impaired microglial phagocytosis, which might be caused by a reduced expression of the GAS6 receptor *MerTK* in primary microglia.

### Myeloid SOCS3-deficiency limits pathological angiogenesis during OIR, but has no effect on physiological vessel formation

**Fig. 3:**
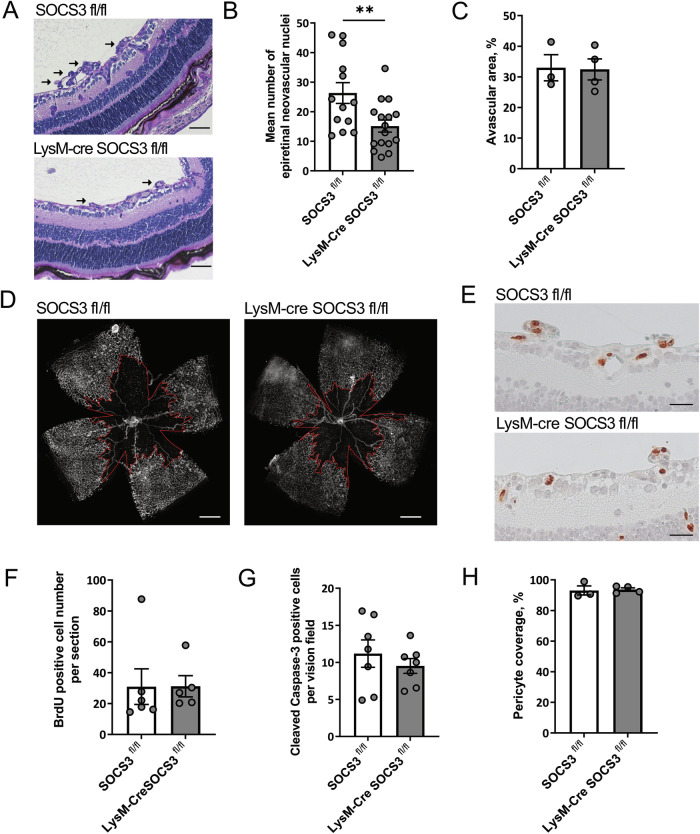
Myeloid SOCS3-deficiency decreases pathological angiogenesis in the OIR model without effects on revascularization. **A**–**H** Myeloid SOCS3-deficient (LysM-Cre SOCS3^fl/fl^) mice or control (SOCS3^fl/fl^) littermates were subjected to the OIR protocol, and sacrificed at P17. In **E**, **F** mice additionally received i.p. BrdU injections at P16. (**A**) Representative pictures of PAS stained retinal cross-sections showing differences in neovascular tufts (black arrows). Scale bar: 50 µm. **B** Quantification of epiretinal neovascular nuclei in PAS stained retinal cross-sections. (*n* = 13–16 mice per group). **C** Analysis of vasoobliteration area demonstrates no differences in the size of avascular area between control and myeloid SOCS3-deficient mice. (*n* = 3–4 mice per group). **D** Representative images of retina whole mounts stained with isolectin B4, which labels retinal vessels in control and myeloid SOCS3-deficient retinas. The red line indicates the location of the avascular area. Scale bar: 500 µm. **E** Representative pictures illustrating no differences in proliferation between control and myeloid SOCS3-deficient mice. Scale bar: 25 µm. **F** Quantitative analysis of proliferation rate showing as number of BrdU-positive cells per retina section. (*n* = 5–6 mice per group). **G** Examination of EC apoptosis in mouse retinas from control and myeloid SOCS3-deficient mice represented as the number of cleaved caspase 3 positive cells in co-localization with retinal vessels (isolectin B4) per vision field (*n* = 7 mice per group). **H** Analysis of vessel pericyte coverage in control and myeloid SOCS3-deficient mice (*n* = 3–4 mice per group). Data are presented as mean ± SEM. **P < 0.01.

Previously, we have demonstrated that SOCS3-deficient microglia had elevated *Gas6* expression, which exhibited enhanced phagocytic activity towards apoptotic ECs, and limited ex vivo angiogenic sprouting [19]. After confirming the occurrence of microglial phagocytosis of ECs in OIR, and the regulation of *Gas6* expression throughout the course of OIR, we engaged mice with myeloid SOCS3-deficiency to investigate pathological angiogenesis during OIR as well as physiological vessel formation. In line with our previous study, our data showed that myeloid SOCS3-deficiency limited the number of pathological vessels in the OIR model. LysM-Cre SOCS3^fl/fl^ mice had fewer epiretinal neovascular nuclei compared to littermate SOCS3^fl/fl^ control mice (Fig. 3A, B). In contrast, physiological vessel formation, which takes place in mice during the initial postnatal weeks, was not affected at P6 and P12 in both animal groups (Fig. S3).

In order to understand the molecular mechanism of how SOCS3-deficiency in myeloid cells regulates pathological angiogenesis in OIR, we assessed at P17 physiological revascularization, evaluated key EC functions such as proliferation and apoptosis, and pericyte coverage of retinal vessels. We observed no difference in the size of the central avascular area between myeloid SOCS3-deficient and control mice, indicating that the physiological revascularization of the retina in the course of the OIR was not affected by myeloid SOCS3-deficiency (Fig. 3C, D). At OIR P17, the number of BrdU+ proliferating cells in retina cross-sections was not altered by myeloid SOCS3-deficiency (Fig. 3E, F). In line, we did not observe altered levels of VEGF, the most potent angiogenic factor stimulating endothelial cell proliferation, in myeloid SOCS3-deficient mice compared to controls (data not shown). Previously, we and others showed that macrophages and microglia can modulate EC apoptosis in the retina [14, 15, 27, 28]. Therefore, we wanted to assess whether myeloid SOCS3-deficiency might limit pathological NV by inducing endothelial apoptosis. However, cleaved Caspase-3 immunostaining in retinal whole mounts revealed no difference in EC apoptosis between retinas of myeloid SOCS3-deficient and sufficient mice (Fig. 3G). Furthermore, myeloid SOCS3-deficiency did not alter pericyte coverage, an indicator for vessel stability and maturation [14, 29] (Fig. 3H).

In conclusion, myeloid SOCS3-deficiency proved to be dispensable for physiological retinal angiogenesis, and did not regulate important vessel parameters. However it limited pathological NV in the mouse OIR model, presumably via the previously demonstrated increase in phagocytosis rate of SOCS3-deficient myeloid cells [19].

### Exogenous GAS6 supplementation increases phagocytosis and limits pathological angiogenesis in the OIR model

**Fig. 4:**
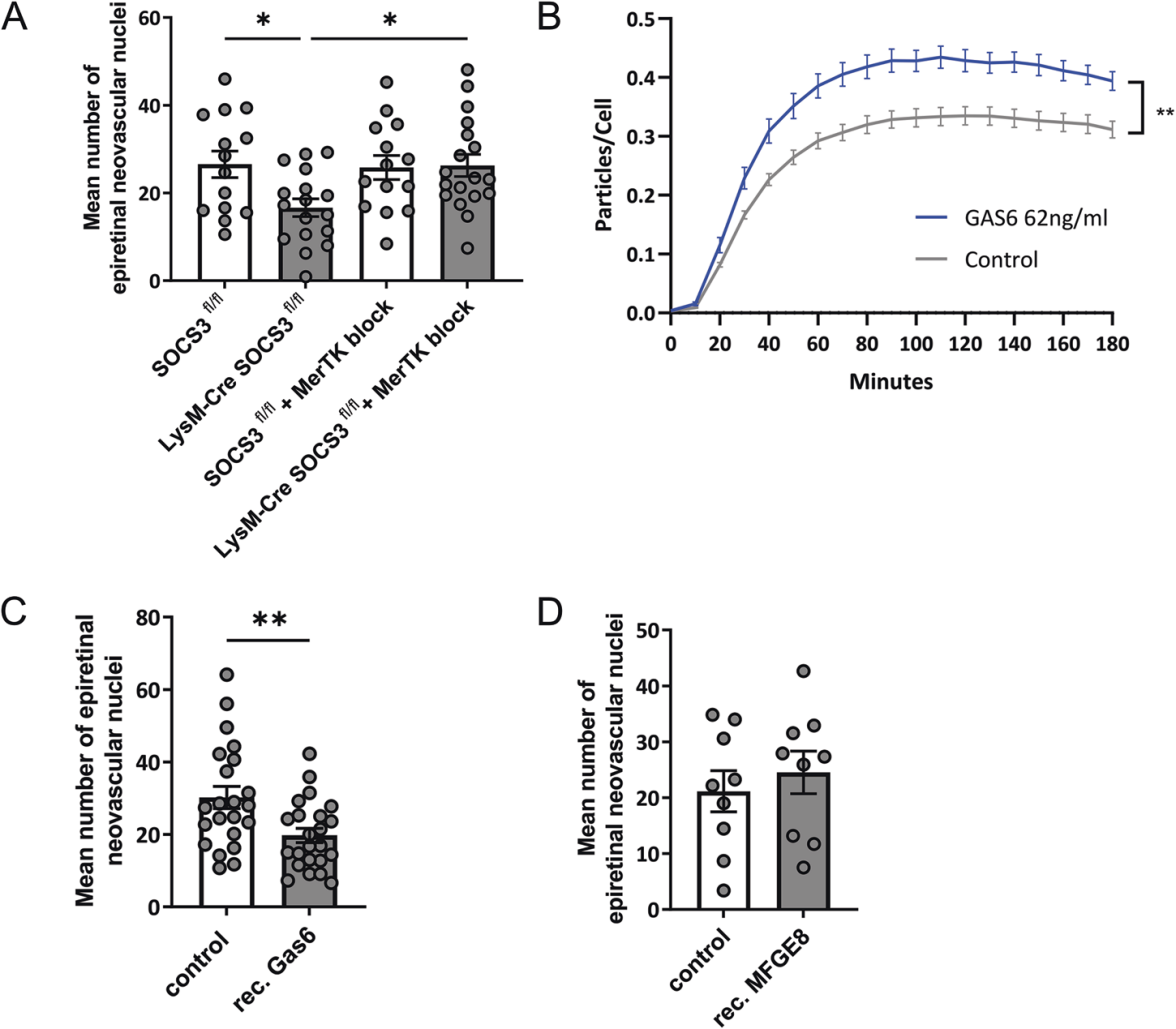
Activation of the GAS6-MERTK pathway decreases pathological angiogenesis in the OIR model. **A** Myeloid SOCS3-deficient (LysM-Cre SOCS3^fl/fl^) mice or control (SOCS3^fl/fl^) littermates were subjected to OIR protocol, injected at P14 intravitreally in the right eye with MerTK-Fc chimera protein, and in the left eye with IgG1 Fc, and sacrificed at P17. The number of epiretinal neovascular nuclei in PAS-stained retinal cross-sections is shown. (*n* = 14–18 mice). **B** Primary microglia were treated with 62 ng/ml recombinant GAS6 for 30 min, followed by a co-incubation with pHrodo-labeled apoptotic HUVECs. Phagocytosis rate was recorded for 3 h with 10 min intervals, and quantified by calculating the mean number of pHrodo particles per microglia. (*n* = 5 microglia cell isolations). **C** C57BL/6 J mice were subjected to OIR, injected at P14 intravitreally in the right eye with recombinant GAS6 protein, and in the left eye with vehicle (PBS), and sacrificed at P17. A number of epiretinal neovascular nuclei in PAS-stained retinal cross-sections are shown. (*n* = 23 mice). (**D**) C57BL/6 J mice were subjected to OIR, injected at P14 intravitreally in the right eye with recombinant MFGE8 protein, and in the left eye with vehicle (PBS), and sacrificed at P17. A number of epiretinal neovascular nuclei in PAS stained retinal cross-sections is shown. (*n* = 9 mice) Data are presented as mean ± SEM, *p < 0.05, **p < 0.01.

To confirm whether the effect of the myeloid SOCS3-deficiency in limiting pathological neovascularization is indeed through a GAS6-dependent manner, we have performed GAS6 neutralization in LysM-Cre SOCS3^fl/fl^ mice and their SOCS3^fl/fl^ littermates by intraocular MerTK-Fc injection. As GAS6 functions as a bridging molecule between PS found on apoptotic cell membranes and MERTK receptor on phagocytes, the presence of excess MerTK-Fc is expected to block GAS6, and consequently PS recognition by phagocytes. As expected, analysis of epiretinal neovascular nuclei at P17 revealed that blocking of GAS6 binding to microglia by MerTK-Fc, reversed the limiting effect of the myeloid SOCS3-deficiency on pathological angiogenesis (Fig. 4A).

To assess the therapeutic potential of recombinant GAS6 in OIR, we first performed real-time in vitro phagocytosis assays in primary microglia pre-treated with GAS6, or PBS vehicle. Our results revealed that recombinant GAS6 supplementation significantly increased the uptake of apoptotic HUVECs by primary microglia (Fig. 4B). Next, we proceeded with an intraocular GAS6 supplementation in WT mice subjected to the OIR protocol. Here, we aimed to overcome the reduction in phagocytosis observed in the early stages of OIR, therefore, we chose P14 as the time point for intervention. Indeed, recombinant GAS6 supplementation at P14 successfully limited the number of epiretinal neovascular nuclear at P17 (Fig. 4C). Additionally, we tested another opsonin molecule, MFGE8, in a similar manner. Surprisingly, MFGE8 supplementation had no effect on the number of epiretinal neovascular nuclei (Fig. 4D), establishing GAS6 as a prominent opsonin in OIR.

These results supported our hypothesis that myeloid SOCS3-deficiency may limit pathological retinal angiogenesis by increased *Gas6* expression and consequent increase in phagocytosis. As exogenous supplementation of GAS6, but not MFGE8, also functions to limit pathological retinal angiogenesis, this molecule is a promising candidate to be further investigated as a therapeutic in IR.

## Discussion

In IRs, immune cells emerged as major regulators in the manifestation and progression for pathological NV. However, we just start to understand the complex crosstalk between immune cells and ECs in retina angiogenesis [1, 9, 30]. Here we identified immune cell phagocytosis as a new crosstalk pathway governing the elimination of altered ECs in the progression of IR. Metabolic, oxidative, and hypoxic alterations in IRs disturb the sensitive retinal homeostasis, resulting in cellular stress and activation of retinal immune cells, especially microglia [1, 30]. In line with multiple previous reports, we observed in the OIR model a change of microglial morphology from a ramified state with long and thin processes, to an ameboid state with larger cell bodies and thicker, shorter processes, indicating microglial activation [1, 4, 24, 25, 31]. In addition, RNA sequencing of OIR microglia confirmed transcriptional activation [25] of genes involved in immune cell proliferation, migration, and several growth factors (e.g., *Vegfa*) were boosted. However, our major interest was the induction of “Phagocytosis” and “Apoptotic Cell Clearance” gene sets in OIR microglia. With endothelial-specific fluorescent reporter mice we could indeed identify microglia cells with engulfed red fluorescent particles originating from ECs in close proximity to the pathological tufts. Interestingly, time course flow cytometry experiments revealed phagocytic microglia also in control mice between P12 and P21. The appearance of EC apoptosis during physiological vessel development is not surprising, as angiogenesis, the major mechanism by which new blood vessels are produced, is a dynamic process tightly linked to local gradients of metabolic and growth factors. Firstly, an excessive number of blood vessels without a mature hierarchical network is formed, requiring, in a second step regression (termed pruning) and removal of excessive ECs [32]. In the second step it has been shown, that EC apoptosis appears within the dynamic process of angiogenesis [32, 33] EC death has been also identified as a major mechanism of programmed regression of transient vessel networks, like the hyaloid vessels and pupillary membrane, that are necessary for the eye development, but redundant when the retina becomes vascularized [34, 35]. Interestingly, in both vessel networks, macrophages are involved in the programmed capillary regression. Also, in stress conditions, like in the hyperoxia-induced vasoobliteration, vessel regression occurs via selective apoptosis of ECs [36, 37]. Surprisingly, in the OIR model, we observed in the retinas at P12 and P15, which is after the vasoobliteration phase, fewer phagocytic microglia, compared to healthy mice. As these time points are coincident with the appearance of retinal hypoxia, due to the lack of sufficient perfusion, the drop in phagocytosis might be explained by our in vitro data, showing that hypoxia treatment of microglial cells reduces the uptake of apoptotic ECs. Based on these data, we hypothesized that the diminished phagocytosis rate might augment the development of the pathological tufts in the OIR, and thus, an activation or induction of EC phagocytosis, might be a new strategy for the treatment of IR. Interestingly, at P17 in the OIR model, when physiological and pathological NV occurs and the retinal tissue is reoxygenized, the phagocytosis rate increased up to 3-fold and stayed elevated until P21 compared to non-OIR controls. To test the relevance of microglial cell phagocytosis on pathological retinal NV, we performed OIR experiments with myeloid SOCS3-deficient mice, as isolated microglia had an increased GAS6-mediated phagocytic activity [19, 20]. In fact, the augmented phagocytic activity reduced the pathological NV in the OIR, and the blocking of GAS6 by an intraocular injection of MerTK-Fc could rescue the effect. Although these are strong data supporting our mechanism, we cannot entirely exclude that myeloid SOCS3-deficient microglia do not have other effects on ECs, as SOCS3 is a crucial negative regulator of cytokine signaling and inflammation [38]. In addition, SOCS3 was also studied in the context of macrophage function and activation, however, some studies showed that SOCS3 is involved in repressing the M1 pro-inflammatory phenotype [39, 40] while others paradoxically reported a reduction in M2 anti-inflammatory phenotype [41, 42]. In addition, SOCS3 was shown to enhance either EC apoptosis, whereas in neurons, SOCS3 over-expression promotes survival [43, 44]. These controversies might explain why, in some studies, conditional knockdown of SOCS3 in ECs, neurons, Müller glia, and in myeloid cells enhanced pathological NV in the OIR model, while we obtained a reduction [45–48]. Thus, the modulation of SOCS3 in myeloid cells might be critical in terms of a therapy in IR patients. To strengthen our hypothesis whether the activation of myeloid cell phagocytosis can be used as a therapy to reduce NV in IR, we performed intraocular GAS6 supplementation in OIR WT mice. As assumed, recombinant GAS6 supplementation reduced pathological NV, and in vitro experiments confirmed an increased phagocytosis rate of GAS6 pre-treated microglia. Consistent in immunofluorescence staining in OIR retinas, GAS6 was present at the EC-microglia interface in the pathological vessels, indicating its opsonin bridging function. In time course experiments with retinal lysates, we observed a reduction of *Gas6* expression at P15, which correlated with a reduction in the percentage of phagocytic microglia. At P17, *Gas6* expression increased, which again correlated with an increase in the percentage of phagocytic microglia. In vitro experiments with hypoxia treatment and/or incubation of microglia with apoptotic ECs revealed a clear hypoxia regulation of *MerTK* expression, the receptor for GAS6, while *Gas6* expression was not regulated. Only the combination of hypoxia and contact with apoptotic ECs reduced *Gas6* expression. Whether these stimuli are relevant in vivo and at which time point in the OIR, needs further investigation. As we did not observe changes in *Gas6* expression in MACS sorted microglia between OIR and controls at the different time points of the OIR, we speculate that the observed changes of *Gas6* expression in whole retina lysates might be the result of an altered *Gas6* gene expression by other cell types, e.g. retinal pigment epithelial cells and photoreceptors [49], or an altered number of immune cells in the retina. Indeed, it has been shown, that myeloid cell numbers increase in the course of hypoxia-triggered proliferative retinopathy in the mouse [1], either due to an increased influx of blood-derived mononuclear cells into the diseased retina [50, 51] or due to an increased proliferation of retinal microglia in the course of OIR [24]. This increased immune cell number, especially at P17, might explain the increased *Gas6* expression in whole retina lysates at this time point. However, further experiments are necessary to determine the source of the altered *Gas6* expression in the whole retina. In addition, it cannot be ruled out that GAS6-independent mechanisms could also contribute to the rate of phagocytosis, such as the degree of vascularization or the rate of endothelial cell apoptosis. Indeed, due to the large avascular area at P12 in the OIR, we observed a lower amount of ECs compared to no OIR retina, and presumably due to the presence of neovascular tufts, a higher number of ECs in OIR at P21 compared to no OIR. This might explain the high phagocytic activity at P21 in the OIR, even though *Gas6* expression is lower, and the lower phagocytic activity at P12 in the OIR, although there is higher *Gas6* expression. Besides the activation of microglial cell phagocytosis, GAS6 might also regulate angiogenesis by other mechanisms, like the inhibition of VEGF-dependent activation of the VEGFR-2, resulting in diminished angiogenesis [52]. However, in vitro treatment of endothelial or microglial cells with recombinant GAS6 did not regulate the expression of VEGF (data not shown).

To test whether other opsonins might have similar anti-angiogenic effects, we applied recombinant MFGE8 in the OIR model. However, MFGE8 supplementation had no effect on retinal NV, indicating that specifically the GAS6-MERTK axis might be a promising target for novel pharmacological intervention strategies in IR. In summary, we provide evidence that the phagocytosis of apoptotic ECs by activated microglia can modulate NV, which extends our knowledge in the complex interaction of microglial cells with ECs in the context of IR.

## Materials and Methods

### Animals

Mice were housed at the Experimental Center at the University of Technology Dresden (Medical Faculty, University Hospital Carl-Gustav Carus) under a 12 h light/dark cycle with access to food and water ad libitum. All animal experiments were reviewed and approved by the Landesdirektion Sachsen (Chemnitz, Germany). WT mice (C57BL/6 J) were purchased from the Janvier Laboratories. Mice with myeloid-specific inactivation of SOCS3 were generated by crossing mice expressing Cre-recombinase under the control of a LysM promoter [53] with SOCS3 flox/flox mice [42]. For experiments presented here, we used mice expressing Cre (LysM-Cre+ SOCS3 flox/flox designated hereafter LysM-Cre SOCS3^fl/fl^) and their Cre-negative littermates (LysM-Cre- SOCS3 flox/flox; designated hereafter SOCS3^fl/fl^). Endothelium-specific fluorescent reporter mice were generated by crossing Ai14 mice ([54] Jackson 007914), a Cre reporter tool strain, with mice expressing Cre-recombinase under the control of an endothelium-specific Flk-1 promoter [55]. Ai14 mice expressing robust tdTomato fluorescence following Flk-1 Cre-mediated recombination (Flk1:Cre/tdTomato flox/flox mice designated hereafter Flk1:Cre/tdTom^fl/fl^) were used for the experiments.

Mice were subjected to the OIR model, as described in several previous studies [14, 18, 21, 56]. Briefly, one-week-old mice (P7) with their nursing mothers were subjected for 5 days to 75% oxygen (hyperoxia), which results in inhibition and regression of the developing retinal vessels (vaso-obliteration). At P12, the mice were returned to room air (21% O_2_). The drop in the oxygen pressure led to the development of hypoxia in the avascular retinal areas, triggering both normal vessel regrowth and pathological neovascularization, which peaks at P17. Mice were sacrificed at P12, P15, P17, or P21, and the eyes were processed. Both genders were used in the experiments. Intravitreal injections were performed with a 33-gauge needle (Hamilton, Bonaduz, Switzerland) under a surgical microscope in anesthetized mice on P14 of the OIR model [14]. Mice received 100 ng of either recombinant mouse MerTK-FC (R&D systems, Minneapolis, Minnesota, USA), recombinant mouse GAS6 (R&D systems), or recombinant mouse Milk fat globule-EGF factor 8 protein (MFGE8; R&D systems) into the right eye. As controls, an isotype-control IgG1 Fc (for MERTK) or PBS (for GAS6 and MFGE8) was injected into the left eye of the same animal. The number of mice used in each experiment is stated in the respective figure legend.

### Histology

To quantify the pathological neovascularization, mice subjected to the OIR model were sacrificed at P17, and eyes were processed for the quantification of epiretinal neovascular nuclei as described previously [14, 21, 56]. Paraffin-embedded serial eye sections of 4 μm thickness were stained with periodic acid-Schiff (PAS) and hematoxylin. Ten to twelve intact sections of equal length (containing the optic nerve region) were evaluated per eye. Retinal vascular cell nuclei anterior to the internal limiting membrane were counted in each section. The mean number of epiretinal neovascular nuclei was calculated by taking the average of the number of counted nuclei per section of each eye. Counting was performed in a blinded fashion. To label proliferating cells in the mouse retina, we performed at P16 intraperitoneal (i.p.) 5-bromo-2-deoxyuridine (BrdU) injections (0.1 mg/1 g of mouse weight) in mice subjected to the OIR protocol, as described [14, 57]. Paraffin-embedded eye sections were de-waxed, and antigen retrieval was performed by boiling the sections in citrate buffer pH 6 (1.8 mM citric acid monohydrate and 8.2 mM sodium citrate dehydrate in dH_2_O). To denature DNA, slides were incubated in 2 M HCl for 30 min at room temperature (RT). Then HCl was neutralized via incubation in 0.1 M Na_2_B_4_O_7_ for 5 min at RT. Endogenous peroxidase was inhibited by Peroxidase Blocking Reagent (Dako, Glostrup, Denmark) in a dilution 1:8 (in PBS) for 10 min. After this step, sections were incubated in blocking buffer from the Vectastain ABC kit (rat IgG) for 20 min at RT. Thereafter, anti-BrdU antibody (1:40, Abcam, Cat. ab6326) was added overnight at 4°C. Following antibody incubation, sections were probed with biotinylated antibody and ABC reagent from the Vectastain ABC kit (rat IgG, Vector Laboratories, Newark, California, USA) according to the manufacturer’s instructions. As a final step, AEC substrate (Vector Laboratories) was applied, and the color development was controlled under the microscope. Slides were counterstained with hematoxylin and mounted with permanent mounting medium. Ten to fifteen retina sections per eye were imaged for quantification using a Zeiss Axiovert 200 M microscope. Results were presented as the average number of BrdU-positive cells per retina section. To estimate the pericyte coverage index of retinal vessels, mice subjected to the OIR model were sacrificed at P17, and eyes were fixed in 4% paraformaldehyde solution (PFA) for 4 h, thereafter treated with 30% sucrose overnight, and then embedded in OCT (VWR International, Darmstadt, Germany). Then, serial 8-µm sections were cut with a cryostat. On the next step, slides were washed with PBS and incubated in blocking solution (5% horse serum, in PBS with 0.5% Triton-X 100) for 20 min at RT. Thereafter, a rabbit antibody against NG2 (1:100, Millipore, Burlington, Massachusetts, USA, Cat. AB5320) and a rat antibody against PECAM-1 (1:25, BD Biosciences, Franklin Lakes, New Jersey, USA, Cat. 550274) were applied at 4°C overnight. After washing, slides were consecutively incubated with anti-rabbit (1:350, Alexa Fluor 594, Invitrogen, Waltham, Massachusetts, USA, Cat. A11012) and anti-rat (1:350, Alexa Fluor 488, Invitrogen, Cat. A21470) secondary antibodies for 2 h at RT. Finally, sections were counterstained with DAPI for 10 min at RT and mounted with fluorescence mounting medium. All PECAM-1-positive structures associated with NG2-positive cells were counted as pericyte-covered vessels. Four to five retina sections per eye were examined using a Zeiss Axiovert 200 M microscope at 20x magnification. The number of pericyte-covered vessels was divided by the total number of vessels in each retina section [14, 58]. For double staining of GAS6 and PECAM-1 or IBA1, cryo-sections (30 µm thick, permeabilized in 0,5% Triton X100 in PBS and blocked with 5% horse serum and 0,5% Triton X100 in PBS) were incubated with a goat antibody against GAS6 (1:100, Invitrogen, Cat. PA5-47981) and a rat antibody against PECAM-1 (1:25, BD Biosciences, Cat. 550274) or a rabbit antibody against IBA1 (1:150, Wako, Osaka, Japan, Cat. 019-19741) in blocking solution (5% horse serum, in PBS with 0.3% Triton-X 100) at 4°C overnight. After washing, slides were consecutively incubated with an anti-goat (1:350, Alexa Fluor 647, Invitrogen, Cat. a21447) and an anti-rat (1:350, Alexa Fluor 488, Invitrogen, Cat. A21208) or with an anti-rabbit (1:350, Alexa Fluor 488, Invitrogen, Cat. A21206) secondary antibody for 3 h at RT.

### Retinal Wholemounts

Analysis of physiological retinal development, EC apoptosis, microglial activation, and phagocytosis was performed using retinal wholemounts. Mice were sacrificed at days 6 and 12 to assess physiological vessel formation, and mice subjected to the OIR model were sacrificed at P17 to assess EC apoptosis and microglial activation, and phagocytosis. Eyes were enucleated and fixed in 10% formalin for 20 min at RT. Afterwards, retinas were extracted, permeabilized, and blocked in PBS with 1% BSA, 0.5% TritonX-100, and 5% goat serum at 4 °C overnight. Next, they were stained with FITC-conjugated Bandeirea Simplicifolia isolectin B4 (Sigma-Aldrich, ST. Louis, Missouri, USA) at a concentration of 10 µg/ml at 4 °C overnight. To assess EC apoptosis, retinas were additionally incubated with antibodies against cleaved caspase 3 (1:100, Cell Signaling Technology, Danvers, Massachusetts, USA, Cat. 9664) at 4 °C overnight, followed by incubation with secondary anti-rabbit antibodies (1:400, Alexa Fluor 594, Invitrogen, Cat. A11012) for 2 h at RT. To assess microglial activation and phagocytosis, Flk1:Cre/tdTom^fl/fl^ mice were used. Here, the isolectin staining step was omitted, instead, the retinas were incubated with rabbit antibodies against IBA1 (1:150, Wako, Cat. 019-19741) and thereafter with secondary anti-rabbit antibodies (1:350, Alexa Fluor 647, Invitrogen, Cat. A21244). Finally, the retinas were flat-mounted with a water-based fluorescent mounting medium and coverslipped. The images were obtained with a Zeiss Axiovert 200 M, or with a Zeiss LSM980 confocal microscope using a 20X water immersion objective. All cleaved caspase-3 positive cells co-localized with retinal vessels (stained with isolectin B4) were defined as apoptotic ECs, and were counted per vision field [59–61]. Analysis of developmental angiogenesis and vaso-obliteration was performed using AngioTool version 0.6a software (to quantify total vessel length and the number of junctions) and Fiji (to quantify avascular area) [62, 63]. Maximum intensity projections and orthogonal views were created using ZEN 3.6 software (Zeiss, Oberkochen, Germany).

### Flow Cytometry

To analyze the rate of phagocytosis throughout the course of the OIR model, Flk1:Cre/tdTom^fl/fl^ mice subjected to the OIR protocol or controls were sacrificed at P12, P15, P17, and P21. Their retinas were isolated and finely chopped using razor blades. The chopped tissue was then enzymatically digested using a solution consisting of either 1 mg/mL dispase II (Sigma-Aldrich), 1.5 mg/mL collagenase D (Roche, Basel, Switzerland), 0.1 mg/mL DNAse I (Roche) in RPMI medium (Gibco, Waltham, Massachusetts, USA), or 31.25 µg/mL Liberase TM (Roche) and 0.1 mg/mL DNAse I (Roche) in RPMI medium (Gibco), and incubated for 20 min at 37 °C, under continuous shaking. The obtained cell suspensions were filtered through 50 µm pore size cell strainers and washed once with FACS buffer (0.5% BSA, 2 mM EDTA in PBS). Cells were stained with anti-CD45-BV711 (Biolegend, San Diego, California, Cat. 103147) antibodies for 1 h on ice, and examined using a FACS Fortessa flow cytometer (BD Biosciences). Data analysis was performed using FlowJo™ Software (v10.9, BD Life Sciences, Ashland, Oregon, USA).

### Magnetic Activated Cell Sorting (MACS) of retinal microglia

Single cell suspensions were obtained as described above for the flow cytometry experiments. The cell suspensions were labeled with CD11b (Microglia) MicroBeads, human and mouse (Miltenyi Biotec, Bergisch Gladbach, Germany, Cat. 130-093-634) at a dilution of 1:100 for 1 h at 4 °C. Next CD11b^+^ cells were purified by applying them to magnetic columns. The purified cells were pelleted by centrifugation (at 500 x *g* for 5 min) and prepared for RNA extraction using the NucleoSpin RNA XS micro kit (Macherey Nagel, Düren, Germany).

### Cell culture and treatments

Primary microglial cell isolation was performed as adapted from a previously described protocol [19, 64]. Briefly, mice aged between P14–P17 were sacrificed, their brains were aseptically removed, and finely chopped using razor blades. Enzymatic dissociation was then performed using a solution containing 0.1 mg/mL papain (Sigma-Aldrich), 1 mg/mL collagenase type I (Thermo Scientific, Waltham, Massachusetts, USA), 1.2 U/mL dispase II (Sigma-Aldrich), 1 mM L-cysteine (Sigma-Aldrich) and 0.1 µg/mL DNAse I (Roche) in DMEM high glucose medium (Gibco) for 40 min at 37 °C under continuous shaking. Afterwards, the cell suspensions were filtered through 100 µm pore size cell strainers, and pelleted by centrifugation at 300 *g* for 10 min. The isolated cells were washed once with 20% fetal bovine serum (FBS) in PBS, then plated on Poly-L-Lysine (PLL) (Sigma-Aldrich) pre-coated tissue culture flasks in microglia growth medium, which consisted of DMEM:F12 supplemented with 20% FBS, 100 μg/ml streptomycin, 100 U/ml penicillin and 5 ng/mL GM-CSF (PeproTech, Cranbury, New Jersey, USA). Cells were cultured at 37 °C in a humidified 5% CO_2_ incubator, refreshing the growth medium three times a week, until a feeder layer was formed. Thereafter, floating microglial cells were collected three times a week and seeded on PLL-coated cell culture surfaces for experiments.

Human umbilical vein ECs (HUVEC) were freshly isolated from human umbilical cords collected from the obstetrics and gynecology department, University Hospital Dresden, after obtaining consent from the donor, according to Jaffe et al. [65] and were maintained at 37°C in a humidified 5% CO_2_ incubator in EC growth medium (EGM). Ethics approval was granted by the ethics committee of the Medizinische Fakultät Carl Gustav Carus of the Technische Universität Dresden (permission EK 203112005). Apoptosis induction in HUVEC was performed by using the natural metabolite staurosporine (Sigma-Aldrich) [66]. HUVECs, after reaching >80% confluency, were treated for 16 h with staurosporine diluted in EGM at a concentration of 200 nM. After treatment, both adherent and floating cells were collected. Cells were washed once with PBS and used in further experiments.

For gene expression analysis under hypoxic/apoptotic stimuli, microglia cells were seeded on 12 well plates at 1×10^6^ cells per well density. The next day, an approximately equal number of apoptotic HUVECs were added on top. Wells containing microglia without apoptotic HUVECs were used as control. Plates were then immediately placed into a hypoxia incubator (1% O_2_, 94% N_2_, 5% CO_2_) for 24 h. An identical plate cultured in a humidified 5% CO_2_ incubator served as the normoxia control.

In vitro phagocytosis experiments were performed by adapting a previously described method [67]. The apoptotic HUVECs were prepared as described above, and were labeled with pHrodo Red SE (Invitrogen) dye at 1 µM concentration in PBS supplemented with 3% FBS for 1 h at RT. Microglia were seeded on 96-well plates at 4 × 10^5^ cells/well density in microglia growth medium. To analyze the effect of hypoxia and presence of apoptotic cells on phagocytosis, microglia cells were seeded in 96-well plates, and were cultured in a hypoxia incubator (1% O2, 94% N2, 5% CO_2_) with apoptotic HUVECs added or not, for 24 h. An identical plate cultured in a humidified 5% CO_2_ incubator was used as normoxia control. To measure the effect of GAS6 on phagocytosis, cells were treated with 62 ng/ml recombinant GAS6, or an equal volume of vehicle PBS in microglia growth medium without GM-CSF, supplemented with 15 mM HEPES for 30 minutes. At the end of GAS6 or hypoxia treatment, microglia were stained with 1.62 µM Hoechst 33342 (Invitrogen), and four images per well were taken with a Cytation 1 device (Agilent, Santa Clara, California, United States). Following, pHrodo Red-labeled apoptotic HUVECs were added on top at 1: 2.5 (microglia: apoptotic HUVEC) density. In the experiments with hypoxia, the plates were returned to the hypoxia or normoxia incubator, following 90 minutes of incubation, an endpoint measurement was performed by taking four images per with the Cytation 1 device. In the experiments with GAS6, kinetic imaging of phagocytosis was performed, by taking four images per well with 10 minute intervals after addition of apoptotic HUVECs, over a duration of 3 hours with the temperature set at 37 °C in the Cytation 1 device. The image stitching and particle counting were performed using the Gen5 software (Agilent). Phagocytosis rate was quantified as the mean number of pHrodo Red particles per microglia, by dividing the total number of pHrodo Red particles by the number of Hoechst 33342-positive microglia counted prior to apoptotic HUVEC addition.

### Cytokine array

Quantification of GAS6 protein levels was performed in retina lysates collected from WT mice subjected to the OIR model and age-matched controls sacrificed at P17. For each cytokine array, three to four retinas from one breeding were pooled. Samples were processed according to the manufacturers’ instructions (Abcam, Cambridge, UK). A total of 200 µg of protein was applied to each membrane. Arbitrary values of GAS6 abundance were calculated as integrated densities of each dot plot normalized by the reference spots. Integrated densities were measured using the Fiji software [63].

### RNA extraction, cDNA synthesis and qPCR

RNA from whole retinas was isolated using TRIzol reagent (Thermo Fisher) according to manufacturer’s instructions. RNA isolation from cultured cells and MACS-sorted cells was performed using a NucleoSpin RNA XS micro kit (Macherey Nagel) according to manufacturer’s instructions. Next, cDNA synthesis was performed using the iScript cDNA Synthesis Kit (BioRad, Hercules, California, USA) according to the manufacturer’s protocol. Expression levels of genes of interest were quantified by performing qRT-PCR’s, using the SsoFast EvaGreen supermix (BioRad) according to the manufacturer’s instructions in a CFX384 Touch Real-Time PCR Detection System (BioRad). Analysis of relative gene expression was performed using 2^-ΔΔCt^ method as previously described [68]. TATA-binding protein (*Tbp*) was used as a housekeeping gene. Primer sequences: *Tbp* forward: 5’-TCTACCGTGAATCTTGGCTGTAAA-3’; *Tbp* reverse: 5’-TTCTCATGATGACTGCAGCAAA-3’; *Gas6* forward: 5’-TGCTGGCTTCCGAGTCTTC-3’; *Gas6* reverse: 5’-CGGGGTCGTTCTCGAACAC-3’; *MerTK* forward: 5’-TCCTTTTGCTGCAGTCACAC-3’; *MerTK* reverse: 5’-TCAGCTGGCTTCACATCAGA-3’; *Glut1* forward: 5’-CATTCTGGCCGAGCTGTTC-3’; *Glut1* reverse: 5’-CGCACAGTTGCTCCACATAC-3’; *Vegfa* forward: 5’-ATGCCCATGAAGTGATCAAGTTCA-3’; *Vegfa* reverse: 5’-ATCCGCATGATCTGCATGG-3’.

### RNA sequencing and bioinformatic analyses

A total of three OIR and three control MACS-sorted microglia samples from mice sacrificed at P17 were analyzed using RNA sequencing (RNAseq). Isolated total RNA (2 ng) was subjected to cDNA synthesis by denaturing the RNA for 3 minutes at 72°C combined with 2 µl of a primer mix (5 mM dNTP (Invitrogen), 0.5 uM dT-primer (C6-aminolinker-AAGCAGTGGTATCAACGCAGAGTCGAC TTTTTTTTTTTTTTTTTTTTTTTTTTTTTTVN, N: a random base V: any base beside thymidine), 4 U RNase Inhibitor (NEB, Ipswich, Massachusetts, USA)). The reverse transcription was performed at 42°C for 90 min after filling up to 10 µl with RT buffer mix for a final concentration of 1x superscript II buffer (Invitrogen), 1 M betaine, 5 mM DTT, 6 mM MgCl2, 1 µM TSO-primer (AAGCAGTGGTATCAACGCAGAGTACATrGrGrG, where rG stands for ribo-guanosine), 9 U RNase Inhibitor and 90 U Superscript II. After cDNA synthesis, the reverse transcriptase was inactivated at 70°C for 15 min. With 10% of the material, a qPCR on full-length cDNA was performed with universal primers (AAGCAGTGGTATCAACGCAGAGT) to determine the optimal number of cycles to avoid under- or overamplification of the samples (for 22 cycles with the below-mentioned protocol). The single-stranded cDNA was amplified using 1x Kapa HiFi HotStart Readymix (Roche) containing 0.1 µM UP-primer*3 under following cycling conditions: initial denaturation at 98 °C for 3 min, 12 cycles (98 °C 20 sec, 67 °C 15 sec, 72 °C 6 min) and final elongation at 72 °C for 5 min. Amplified cDNA was purified using 0.6x volume of hydrophobic Sera-Mag SpeedBeads (GE Healthcare, Chicago, Illinois, USA) rebuffered in a buffer consisting of 10 mM Tris, 20 mM EDTA, 18.5% (w/v) PEG 8000, and 2 M sodium chloride solution. The cDNA was eluted in 12 µl nuclease-free water, and quality and concentration were determined with the Fragment Analyzer NGS Kit (Agilent). For library preparation, 2 µl of amplified cDNA was tagmented at 55 °C for 15 min in a total volume of 4 µl containing 1x Tagment DNA Buffer and 0.8 µl Tagment DNA Enzyme (from the Illumina DNA Prep - Tagmentation Kit, Illumina). The reaction was stopped by adding 1 µl 0.1% SDS and incubation for 15 min at 37 °C. After removing the supernatant, the samples were resuspended in 1x concentrated KAPA HiFi HotStart Ready Mix and 0.7 µM dual indexing primers and PCR run under the following conditions: (72°C 3 min, 98 °C 30 sec, 12 cycles (98 °C 10 sec, 63 °C 20 sec, 72 °C 1 min), 72 °C 5 min). After PCR, libraries were purified with 0.9x volume of rebuffered Sera-Mag SpeedBeads (GE Healthcare), followed by a size selection with 0.6x (right side) and 0.9x (left side) volume of Sera-Mag SpeedBeads to get a fragment size distribution between 200–700 bp. The libraries were quantified with the Fragment Analyzer NGS Kit, and sequenced with a Novaseq 6000 system (Illumina) on a S4 flowcell in 100 bp paired-end XP mode, aiming at a minimum sequencing depth of 35 million reads per library.

FastQC (http://www.bioinformatics.babraham.ac.uk/) was used to run a basic quality control of the resulting sequencing data. Adapters (Nextera: CTGTCTCTTATA) and poly(A/T) tail sequences were trimmed with cutadapt (v4.4) [69], and only pairs with a minimum length of 35 bp for both reads were kept for further analysis. Fragments were aligned to the mouse reference genome GRCm39 with support of the Ensembl 104 splice sites using the aligner STAR (v2.7.10b) [70]. Counts per gene and sample were obtained based on the overlap of the uniquely mapped fragments with the same Ensembl annotation using featureCounts (v2.0.1) [71]. Normalization of raw fragments based on library size and testing for differential expression between the different cell types/treatments was done with the DESeq R package (v1.38.3) [72].

To identify differentially expressed genes, counts were fitted to the negative binomial distribution, and genes were tested between conditions using the Wald test of DESeq2. Resulting *p*-values were corrected for multiple testing with the Independent Hypothesis Weighting package (IHW 1.12.0) [73]. Genes with a maximum of 1% false discovery rate (FDR 1) (padj ≤ 0.01) were considered as significantly differentially expressed.

GSEA was performed with the GSEA software (Broad Institute) [74] with following key parameters: number of permutations: 1000; minimum term size: 15; maximum term size: 500. Gene Ontology: Biological Processes (GO:BP) gene set from Molecular Signatures Database (MSigDB) [75] was used as input.

### Statistical analysis

The statistical analysis was performed, and graphs were drawn using GraphPad Prism 10 software (Boston, Massachusetts, USA). Data are presented as the mean ± standard error of the mean (SEM). The statistical significance of data was determined using unpaired 2-tailed Student’s t-test for single comparisons, and 1-way ANOVA test with Tukey test for multiple comparisons. In the time-dependent in vitro phagocytosis assay 2-way ANOVA was applied. In the case of normalized parameters, here in the cytokine array, a one-sample t-test was applied.

## Supplementary information

Supplementary figures

## Data Availability

The RNA sequencing data are deposited at zenodo.org (accession ID: 13759119; databank URL: 10.5281/zenodo.13759119). Further data are available upon request from the authors.
