# Supplementary material for: Modulation of microglial phagocytosis via the GAS6-MERTK pathway regulates pathological angiogenesis in the mouse oxygen-induced retinopathy model
Source: Cell Death Dis. 2025 Jun 2;16(1):428. doi: 10.1038/s41419-025-07744-4 (PMC12130206; doi:10.1038/s41419-025-07744-4)
Supplement: Supplementary file 1 — Supplementary figures [file 41419_2025_7744_MOESM1_ESM.pdf]

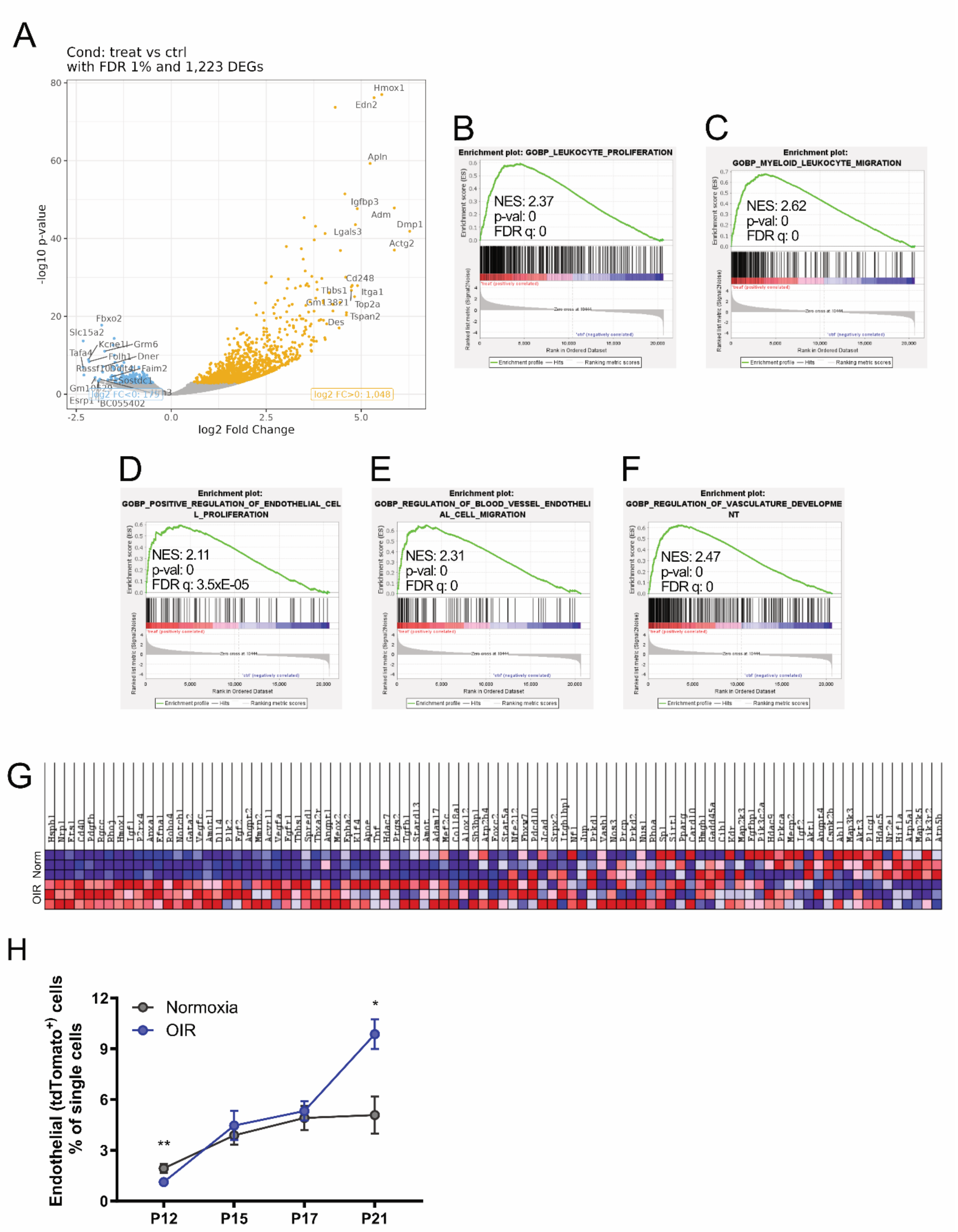

Fig. S1 Transcriptomic changes in OIR microglia and percentage of endothelial cells in the course of the OIR.

Bulk RNA sequencing was performed on microglial cells (CD11b^{+}) MACS sorted from C57BL/6J mice subjected to the OIR protocol, or littermates kept in room air, and sacrificed at P17. **(A)** Volcano plot

of differentially expressed genes at FDR1. **(B-F)** GSEA results are shown for Leukocyte Proliferation **(B)**, Myeloid Leukocyte Migration **(C)**, Positive Regulation of Endothelial Cell Proliferation **(D)**, Regulation of Blood Vessel Endothelial Cell Migration **(E)**, Regulation of Vasculature Development gene sets **(F)**. **(G)** Heatmap of genes found on Regulation of Blood Vessel Endothelial Cell Migration gene set. **(H)** Flk1:Cre/tdTom$^{fl/fl}$ mice subjected to OIR, or kept in room air were sacrificed at P12, P15, P17, and P21, and flow cytometry was performed on retinal cell suspensions to assess the percentage of endothelial cells (tdTomato^{+}). The frequency of endothelial cells is shown as a % of all single cells. (n = 6 - 11 mice at P12, n = 10 - 11 mice at P15, n = 6 - 11 mice at P17 and n = 3 - 5 mice at P21). Data are presented as mean ± SEM, *: $p < 0.05$, **: $p < 0.01$

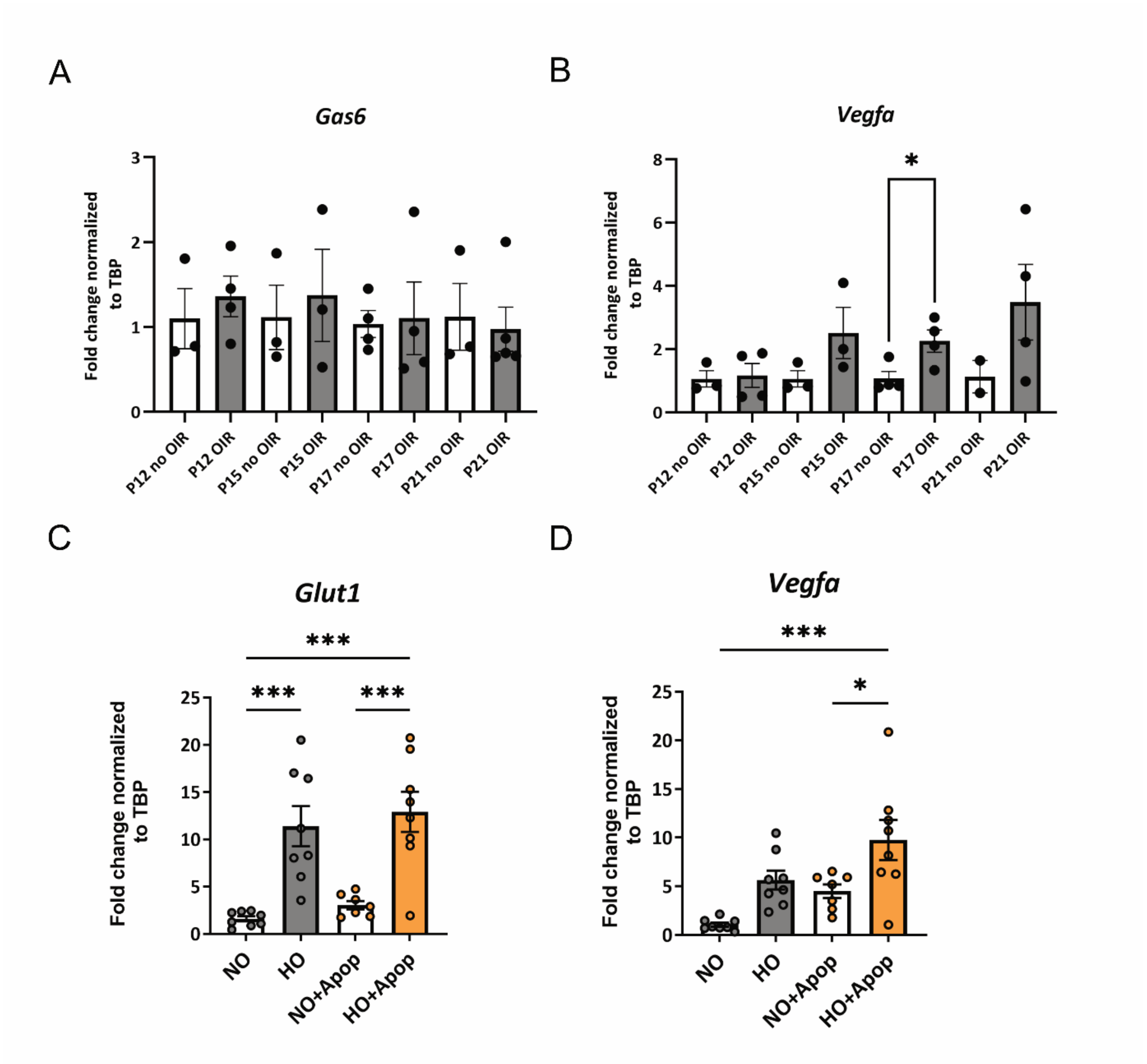

Fig. S2 Gene expression of in MACS-sorted microglia from OIR and controls and of microglia under hypoxia.

(A, B) Expression of *Gas6* and *Vegfa* in MACS-sorted microglia between OIR and controls at P12, P15, P17 and P21. n = 2 – 5. **(C, D)** Analysis of *Glut1* and *Vegfa* mRNA expressions in primary microglia cultured with or without apoptotic HUVECs under normoxia or hypoxia (1% O2), for 24 h. NO: normoxia, HO: hypoxia, Apop: apoptotic HUVECs. n = 6 – 8 microglial isolations. Data are presented as mean ± SEM, *: $p < 0.05$, ***: p

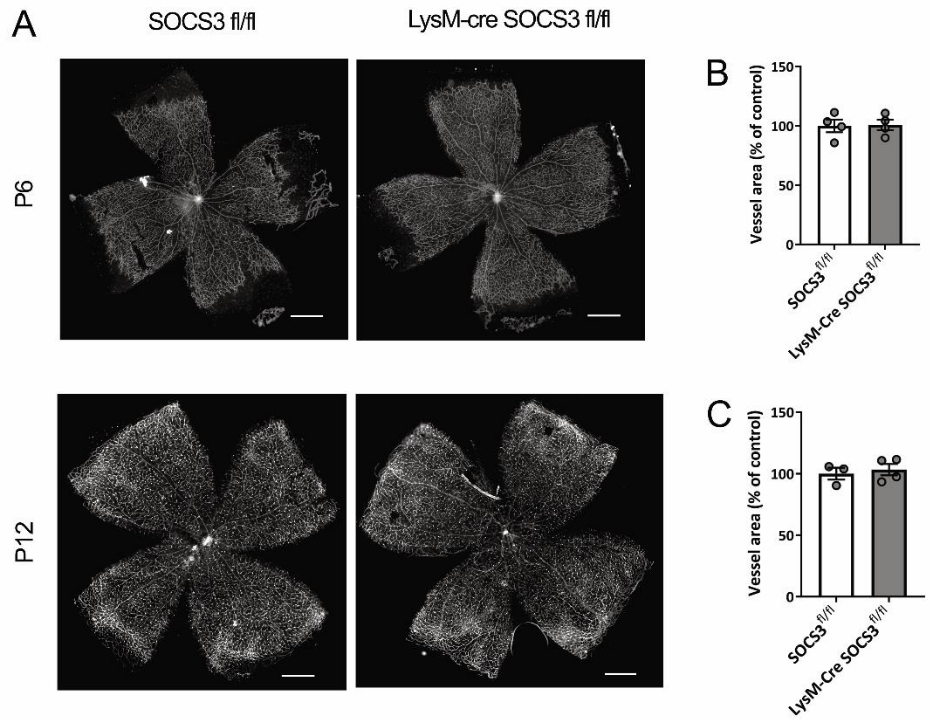

Fig. S3 Myeloid SOCS3-deficiency does not affect physiological retinal angiogenesis.

(A) Representative images of physiological P6 and P12 retinas stained with isolectin B4. Scale bar: 500 µm. **(B, C)** Corresponding quantifications demonstrating no differences in the total vascular area between control and myeloid SOCS3-deficient littermates at P6 **(B)** and P12 **(C)** (n = 3 – 4 mice per group) Data are presented as mean ± SEM.
